# Advancing evidence-informed practice in modern zoos: research priorities for animal welfare, conservation, and social legitimacy

**DOI:** 10.3389/fvets.2026.1793169

**Published:** 2026-04-16

**Authors:** Paul Rose, Idu Azogu-Sepe, Lisa Clifforde, Jessica Harley, Kerry Hunt, Robert Kelly, Ricardo Lemos de Figueiredo, Justine Partoon, Christina R. Stanley, Mark Whiteside

**Affiliations:** 1 Centre for Research in Animal Behaviour, Department of Psychology, University of Exeter, Exeter, United Kingdom; 2 WWT Slimbridge Wetland Centre, Slimbridge, United Kingdom; 3 Serengeti-Park Department of Research, Serengeti-Park Hodenhagen GmbH, Hodenhagen, Germany; 4 Zoological Society of London, London, United Kingdom; 5 Knowsley Safari, Prescot, United Kingdom; 6 SEA LIFE, Poole, United Kingdom; 7 Bristol Zoological Society, Bristol, United Kingdom; 8 Zoos South Australia, Adelaide, SA, Australia; 9 Animal Behaviour and Welfare Research Group, School of Natural Sciences, University of Chester, Chester, United Kingdom; 10 School of Biological and Marine Sciences, University of Plymouth, Plymouth, United Kingdom

**Keywords:** applied welfare research, evidence-based management, ex situ conservation, human-animal interactions, social license to operate, zoo animal welfare

## Abstract

Modern zoos operate at the intersection of conservation responsibility, ethical accountability and scientific advancement, while facing increasing public scrutiny and rising expectations for transparency and measurable impact. This scrutiny often focuses on animal welfare and the justification for maintaining wild animals in captive environments. Consequently, there is a growing need for zoo-based research to move beyond often anecdotal practice toward robust, evidence-informed decision-making. Evidence refers to systematically collected data and critically evaluated research used to inform management strategies that promote the zoo’s key aims. This article synthesises key areas in which contemporary zoo operations rely on limited or untested evidence and identifies priority research questions critical to the future legitimacy and effectiveness of zoos. The paper explores several interlinked themes: the welfare implications of reproduction and breeding management; the role of social bonds in welfare, stress resilience, and translocation outcomes; the underrepresentation of nutrition within animal welfare research; the impacts of human–animal interactions involving both staff and visitors; the maintenance of species-specific behaviours and behavioural diversity in ex situ managed populations; and the relationship between animal welfare, visitor experience, and a zoo’s social licence to operate. These themes were identified through persistent assumptions in zoo practice, gaps in the empirical literature, and increasing societal expectations for transparency, ethical justification, and measurable outcomes. Across these areas, the paper highlights a recurring challenge: management practices are frequently justified on theoretical or historical grounds rather than validated, applied outcomes, and conclusions from single studies are often overinterpreted despite limited replication or contextual relevance. Our article therefore emphasises the importance of defining credible evidence, critically appraising published research, and adopting replicated, collaborative multi-institutional and mixed-methods approaches. By outlining these research priorities, this paper provides a framework for advancing zoo-based science that can directly inform management decisions, improve animal welfare outcomes, strengthen conservation impact, build public trust and thus future-proof the zoo industry. Ultimately, we argue that the future success of zoos depends on aligning ethical practice, rigorous research, and meaningful visitor engagement through transparent, evidence-driven approaches.

## 1 Introduction

The modern age presents zoos with a complex and evolving mandate (1), moving beyond their origins as living collections to become pivotal centres for conservation, education, engagement and wellbeing (human and animal) (2, 3). Increasing public scrutiny and the global biodiversity crisis necessitates a profound re-evaluation of the research questions zoos and aquariums (hereafter “zoos”) prioritise. Research involving zoos enables knowledge gain and meaningfully expands our understanding of species biology (4–6). To truly enhance the impact, quality and application of their science, zoos must reflect critically on the questions they are asking (7, 8). Alongside this, zoo-focused research should diversify the types of question asked and subject(s) chosen for such investigation (9–12). In this article, we argue that “needed research” in the contemporary zoo context is fundamentally evidence-based, deeply considerate of wider societal impacts and boldly addresses challenging questions to ensure tangible benefits for their animals, their workforce, and their overarching aims and values.

Modern zoos have embraced a multifaceted mission (13), and reputable, scientifically-grounded establishments are integral to global conservation (14), advocacy (15, 16), welfare policy development (17, 18), promoting human wellbeing and connection to nature (19) and scientific research (20), all of which foster broader understanding of biodiversity and the natural world. The modern zoo’s aims of conservation, research, education and entertainment, as described in Kleiman (21)‘s seminal work, are continually evolving, and as we move further into the 21st century we see other features included such as animal welfare and human wellbeing (3). Other voices argue for further evolution to consider the zoo’s wider influences over, for example policy and community action (22). However, as the scope of a zoo’s mission expands, so too does the potential for competing priorities and unintended consequences to arise. Therefore, it is critical that the ethical obligation to ensure the highest possible standard of animal welfare remains paramount and is not compromised by other institutional objectives. The continued relevance and legitimacy of zoos rests on their ability to promote and implement demonstrable commitments to welfare and conservation outputs, moving beyond storytelling and exhibition of living species. Mission-focussed research is not a supplementary activity but a foundational one for a zoo’s existence and wider societal relevance.

As public awareness of the biological needs of captive species grows, zoo-based practices that impact population management (23) and visible signs of animal welfare (24) face increasing scrutiny. Consequently, evidence-based practices (25) are critical for upholding population management and conservation aims. We define evidence-based animal care as the scientific basis for in-zoo housing and husbandry, whereby empirical information on species’ evolutionary history, ecological requirements, and behaviour patterns are systematically embedded into better-practice approaches for management and care. Animal care is part of evidence-based management requiring continual review of husbandry inputs to ensure positive welfare outcomes for individual animals (26). Focusing research to examine aspects of zoo operations (e.g., animal care, population management, welfare assessment, conservation action and visitor engagement and education) helps ensure zoos are seen as credible and scientifically centred organisations.

The core of this paper explores a series of research topics, considered by the authors as important to the progression of zoo research moving forwards. This list of topics is not exhaustive, but one that we feel would have positive impacts on animal management and zoo operations. The paper appraises research’s direct impact on the zoo’s environment, on the animals themselves, and on the zoo’s anthropological elements. By critiquing why these topics (Table 1) are deserving of scrutiny, we aim to provide a roadmap to answer these questions that will allow zoos to maximise their positive impacts on biodiversity conservation, knowledge gain, animal welfare and their social licence to operate. We acknowledge that our broad themes are not mutually exclusive and there are likely areas of crossover between different topics and areas of scrutiny.

**Table 1:** Examples of the key research questions for zoos in the 21st century discussed in this paper.

Broad theme	Research question	Future areas of investigation	Rationale for importance
Animal impacts	What is the role of domestication to the genetic integrity of zoo populations?	How rapidly do domestication-related traits emerge in captive populations? How do these traits influence behavioural competence, welfare, and conservation potential across generations?	Addresses the inherent conflict between adaptations for captive welfare and the retention of wild-type traits essential for reintroduction and long-term conservation.
	What is pragmatic welfare assessment that is feasible to apply?	Which species-specific behavioural, physiological, or cognitive indicators provide reliable and practical welfare assessment tools for zoo animals? How can these be standardised across institutions?	Moves welfare evaluation towards holistic, affective-state-centric, and practical tools that directly inform and improve animal care on an individual and species level. Whilst this topic has institutional impact, we feel the primary outcome relates to the zoo’s animals, even though organisational factors influence their implementation.
	What are the benefits of natural vs. commercial diets, and dietary presentation, for captive wild animals?	How do naturalistic versus formulated diets influence species-typical foraging behaviour, cognitive engagement, and long-term welfare outcomes across different taxa?	Recognises that diet extends beyond nutrition to influence species-specific feeding behaviours that are fundamental to overall welfare and conservation of adaptive potential.
Human impacts	How might human-animal interactions and visitor presence influence animal welfare?	Under what conditions do interactions with keepers or visitors positively or negatively influence behavioural and physiological welfare indicators? How do species traits and individual differences mediate these responses?	Navigates the paradox of visitor engagement as a financial and educational driver versus its potential ethical and welfare risks to animals.
	What are zoo visitor experiences and expectations of their local zoo?	How do visitor perceptions of welfare, conservation, and educational value influence their engagement, learning outcomes, and support for zoos?	Addresses the evolving public demand for tangible conservation and welfare benefits, requiring zoos to understand and actively shape visitor perceptions and engagement.
Environmental impacts	How might social bonds be impacted by translocation for population management?	How do social relationships influence stress responses, group integration, and reproductive success following transfers between institutions?	Critical for minimising stress, ensuring successful social integration, and maintaining species-specific behaviours vital for welfare and potential reintroduction outcomes.
	How can we retain genetic diversity and wild-type behaviours across generations in the zoo?	What husbandry practices and environmental conditions best maintain species-typical behaviours and ecological competence in long-term captive populations?	Essential for producing populations that are not only genetically robust but also behaviourally competent for their ultimate conservation purposes (including those populations deemed for in situ reintroduction).
Institutional and reputational impacts	How can we have reliable evidence for evidence-based approaches?	What criteria define credible evidence for zoo management, and how can collaborative research frameworks improve replication, transparency, and applicability of findings?	Ensures scientific rigour and practical applicability of interventions, moving beyond anecdotal practices to verifiable improvements in animal care and management.
	What are the positive welfare benefits of reproductive opportunities?	Does the opportunity to reproduce improve behavioural diversity, affective state, or overall welfare across different taxa held in zoos?	Adding evidence to anecdote that breeding is perceived to improve welfare. Do species benefit from being able to reproduce?
	How can collaboration improve the impacts of animal welfare science?	How do multi-institutional collaborations and interdisciplinary research networks improve the translation of welfare science into practical husbandry improvements?	Confronts critical implementation gaps between scientific findings and their consistent translation into demonstrable, measurable improvements in daily zoo operations by considering how other disciplines can help.

## 2 Animal impacts

### 2.1 Welfare versus conservation: the role of domestication and its impact on the future of zoo populations

Domestication occurs when animals adapt to humans and a human-created environment (27, 28). Recurring captive management practices cause genetic adaptations over generations or influence developmental mechanisms resulting in changes to biological traits (27, 29). Domestication is typically associated with animals with close ties to humans over many generations, such as seen for agricultural livestock (30). It may, therefore, seem strange to discuss domestication in the context of a modern zoo. However, domestication is complex and with knowledge of the field advancing in recent years (31) its in-zoo impacts may be more profound than originally considered. To understand the impacts of domestication, we need a clear definition of what it is. However, this is not trivial as, despite its long history, there is a lack of consensus (29, 31–33) surrounding a finite definition. Early definitions described a domestic animal as being “bred in captivity for the purpose of economic profit to human community that maintains total control over its breeding, organisation or territory and food supply (34).” However, more recent definitions emphasise the process itself, where animals become gradually adapted to humans and their captive conditions over generations (32). This suggests, for the purpose of this review, a continuum in the degree to which an animal is adapted to a human environment and to which that animal depends on it (35); resulting in changes to genetics along with inherited behavioural, cognitive and physiological characteristics (29, 33, 36). This theory could have major implications for zoos as the process of domestication starts as soon as an animal is transferred to captivity and breeds (32). There are few studies conducted on zoo populations and on the mechanisms driving the early aspects of the domestication process, but research on red (silver) foxes (*Vulpes vulpes*) reveals that expression of phenotypic traits akin to those seen in domestic breeds can occur within a few generations in captivity (37).

For zoo animals, domestication will likely impact two species-centred priorities for modern zoos - welfare and conservation. Assessing the potential welfare and conservation impacts in zoos is constrained by the need to infer from livestock and laboratory animal research. The impact of domestication on welfare is complex. Evidence suggests that domestication helps animals cope better with captivity by reducing fear of humans (38), which can reduce the stress associated with capture, housing, and other agricultural and laboratory practices (39). In addition, thresholds to express behaviour differ in domestic populations (29), these can include increased timidity and reduced aggression (40), some of which can promote welfare in a human-controlled environment. Other outcomes of domestication would promote poor welfare, such as extreme growth rates, reduced fertility, brachycephaly and reduced brain sizes (41–43).

For conservation, domestication has its greatest impact on animals destined for wild release as it causes them to significantly differ from their ancestral lineage and/or wild-reared counterparts (44). For instance, domesticated animals show lower incidences of anti-predation behaviour (45), lower motivation to forage (46) and increased susceptibility to predation when released into the wild (47). Such behavioural deficiencies between released animals and wild reared conspecifics are one of the main reasons why many reintroduction projects fail (48, 49). Additional complications associated with depleted genetic diversity plus behavioural, cognitive and physiological deficiencies make it increasingly difficult for zoos to achieve their conservation objectives (50, 51). However, animals can be primed for release into the wild through environmental enrichment (52) and by provisioning more naturalistic captive environments (53). If, as suggested, domestication within zoos has already begun (32), potentially long-term responses may include processes such as de-domestication and feralisation, whereby animals return to a wild or self-sustaining state through carefully designed “naturalistic” housing over generations (54, 55) but any implementation of such protocols will require thorough ethical debate and further research (56, 57).

Zoo animals are unique compared to livestock and laboratory models that are commonly studied in domestication research in that they, and their offspring, are not always destined for a life completely under human control. In some cases, zoo animals are housed temporarily for release into the wild, for instance a translocation between sites of wild caught individuals or when zoos are asked to provide housing for confiscated individuals (58). Some zoo animals are housed in captivity for multiple generations prior to being involved with reintroduction programmes, perhaps driven by practices like EAZA (European Association of Zoos & Aquaria) Ex situ Programmes (EEPs). Some zoo animals and their offspring similar to the aforementioned livestock and laboratory animals, will be housed in captivity with no chance of being released. This makes management decisions difficult. Do you focus on welfare, or do you focus on conservation, or is there a coincidence of interest where both can be maximised?

We are not ready to make these decisions yet. Despite it being clear that domestication can have a marked influence on animal populations, we need to understand more about the process, particularly during its early phases (59). This needs species specific research and will require carefully designed studies to investigate how selection can influence genetic adaptations and developmental mechanisms and, in turn, how this changes behavioural, cognitive and physiological characteristics. From this we can determine the full impact that domestication may have on both animal welfare outputs and the animal centred conservation potential of these released individuals and their offspring. Such research aids our understanding of any trade-offs to resolve between welfare and conservation (60, 61). Crucially we need such research to inform the community and promote discussion about domestication, its merits and costs, and associated ethical considerations to determine if we should embrace it or mitigate its impact before these become irreversible.

### 2.2 Developing a pragmatic approach to welfare assessment to prioritise species-specific evaluations

Formal welfare assessment is increasingly mandated by legislation, reinforcing the ethical and moral requirement to ensure high in-zoo welfare standards. The Irish Standards of Modern Zoo Practice (62) require zoos to document welfare assessments, while the new UK Standards for Modern Zoo Practice (63), effective from 2027, mandate ethical review and formal assessment of all visitor–animal interactions. Zoo accrediting bodies (e.g., those in the UK, Europe, North America and Australasia) also require members to apply structured frameworks, including the Five Domains Model, and conduct “bespoke formal and comprehensive welfare assessment” (17). Effective welfare assessment is not only a legal expectation but essential for maintaining a zoo’s social licence to operate (64).

The challenge lies in applying welfare assessment across the extraordinary taxonomic diversity managed by zoos. The Zoological Information Management System (ZIMS) holds records for >10 million individuals across over 22,000 species (5). To address this scale, the sector initially prioritised the development of ‘general’ or ‘broad-spectrum’ assessment tools (65, 66). This enabled rapid deployment of formal assessment without the need for bespoke templates for every species. However, this strategy diverges from farm animal welfare science, where initiatives such as Animal Welfare Indicators (AWIN) built on the work of the Welfare Quality® project and developed species-specific tools rather than a single generic framework (67), for species such as goats (*Capra aegagrus hircus*), sheep (*Ovis aries*), donkeys (*Equus asinus*) and turkeys (*Meleagris gallopavo domestica*). Although species-specific assessment tools for zoo-housed animals do exist they remain relatively rare by comparison and focused on charismatic mammals (68–71).

The limitations of broad-spectrum protocols are increasingly evident. Generic assessments require assessors to interpret indicators using detailed knowledge of each species’ natural history and behaviour (72). This shifts the burden of tailoring onto zoo staff, who must conduct extensive background research to ensure accuracy. Such work is often impractical within typical staffing and time constraints; few zoos employ dedicated welfare officers, and operational demands restrict the depth of research staff can undertake (73). Consequently, generic assessments risk invalid or inconsistent results. A more pragmatic approach would front-load the research effort through expert-led development of species-specific templates. Additionally, the limited validation of indicators in generic tools raises concerns about robustness, reliability, and repeatability (74). This lack of specificity can create systematic biases, for instance, mammals often receive higher welfare scores and fewer ‘Not Applicable’ or ‘I Don’t Know’ responses than other taxa (75). These issues conflict with the requirement that welfare assessments be rapid, valid, reliable, and feasible (70, 76). A scoping review similarly concluded that validated species-specific assessments are needed to inform management decisions and promote good welfare outcomes (77).

Although the Five Domains Model is a useful conceptual tool for identifying welfare compromise and enhancement, it faces challenges when used quantitatively. Mental states cannot be directly measured, meaning Domain 5 cannot be conclusively validated (78). Assessments reliant on subjective judgement may therefore have limited repeatability (79). Accurately determining affective experience requires a shift toward species-specific tools and complementary frameworks that incorporate animal agency and welfare enhancement. One example is the ‘Six Cs’ - Coping, Comfort, Choice, Control, Challenge, and Compassion (26), which emphasise agency and positive engagement.

Positive welfare centres on animal agency, the animal’s ability to act, explore, and influence its environment (80, 81). Welfare is increasingly defined by opportunities for animals to perform meaningful, self-directed behaviours rather than by resources alone (80, 82). While this aligns with Domain 4 (Behavioural Interactions) of the Five Domains Model, the Six C’s offer a broader framework for embedding welfare enhancement into assessment. Developing species-specific templates will require coordinated, evidence-led processes, time and money but if well-managed and well-designed, they will be meaningful and adopted for years to come.

A practical starting point is to focus on species with strong best-practice guidelines and research foundations. Collaborative indicator development (through accreditation bodies, taxon advisory groups, and the like) should be embedded into husbandry and care manuals. For example, the Association of Zoos and Aquariums (AZA)‘s ball python (*Python regius*) Welfare Indicator Guide, produced in collaboration with AZA’s Snake Taxon Advisory Group (83) provides an evidence-based compilation of physical and behavioural outputs that serve as essential building blocks for species-specific assessment. Foundational work such as Skovlund, Kirchner (84)‘s review of polar bear (*Ursus maritimus*) indicators, reptile-focused frameworks from Benn, McLelland (85) and Yeh, Huang (86), and Rose (87)‘s giraffe (*Giraffa* sp.) specific welfare acronym illustrate how species-focused welfare assessment metrics can be developed in a similar way to the “gold standards” of bespoke welfare frameworks seen in agriculture. Field-testing these indicators across institutions is essential to ensure reliability and repeatability. Through sustained collaboration and integration of positive welfare frameworks, the zoo community can close research gaps and deliver functional, scientifically robust, species-specific assessment tools that genuinely improve animal welfare outcomes.

### 2.3 The impacts of natural versus commercial diets, and dietary presentation, on adaptive behaviours of captive wild animals

Nutrition is comparatively underrepresented in zoo welfare research, with publications prioritising behavioural and environmental factors, whilst physical domains, such as nutritional management, receive less empirical attention (12, 88). Whilst zoos need to ensure that their animals receive adequate calories and nutrients, just focusing on this when planning diets provides an incomplete perspective. Diet also impacts feeding behaviours, cognitive engagement and broader adaptive capabilities that contribute toward welfare (89–91), highlighting that it is not only a food’s nutritional content, but also the form and function of delivery that has an impact. The Five Domains Model underscores the link between nutrition and behaviour, with nutritional inputs directly influencing behavioural expression, which in turn affects mental state and overall wellbeing. Providing naturalistic diets that resemble the food type, complexity and acquisition challenge seen in the wild can positively impact behaviour. However, such naturalistic dietary items can be nutritionally variable - for example capelin (*Mallotus villosus*) protein content varies between 56 and 76% dry matter and herring (*Clupea* sp.) fat content varies from 16.4 to 38.3% dry matter (92). This demonstrates that while naturalistic diets can enhance behavioural welfare, their nutritional variability necessitates rigorous assessment to ensure they support, rather than compromise, overall animal welfare outputs. In contrast, manufactured (“complete”) diets are designed to meet specific nutritional requirements and are consistent in nutritional composition (across batches) but can fail to encourage natural foraging behaviours fundamental to species typical ecology or success in the wild (61).

There is a growing selection of complete diets available for aquatic species, these are nutritionally reliable with a consistent level of each nutrient. Many complete diets are fortified with essential macronutrients (88) and aquatic diets are often formulated to reduce nutrient leeching in water to maintain water quality (93), further enhancing their nutritional reliability. Fishes exhibit diverse feeding strategies in the wild with grazing, predation and filter-feeding as some examples, these are tied to sensory cues, motor skills, and ecological interactions (94). If diets are reduced to pellets or flakes for in-aquarium nutrition, the complexity of these behaviours can be diminished. Research on aquaculture species shows that feeding regimes that align with natural diet types and patterns are associated with increased natural feeding behaviours improving welfare outcomes (95). For example, endocrine mechanisms controlling feeding appetite and satiation (key controllers of feeding and intake behaviours) are stimulated differently by varied diet types, suggesting that fish are behaviourally sensitive to diet composition (95) and therefore this needs to be factored into captive nutritional protocols.

**Figure 1:**
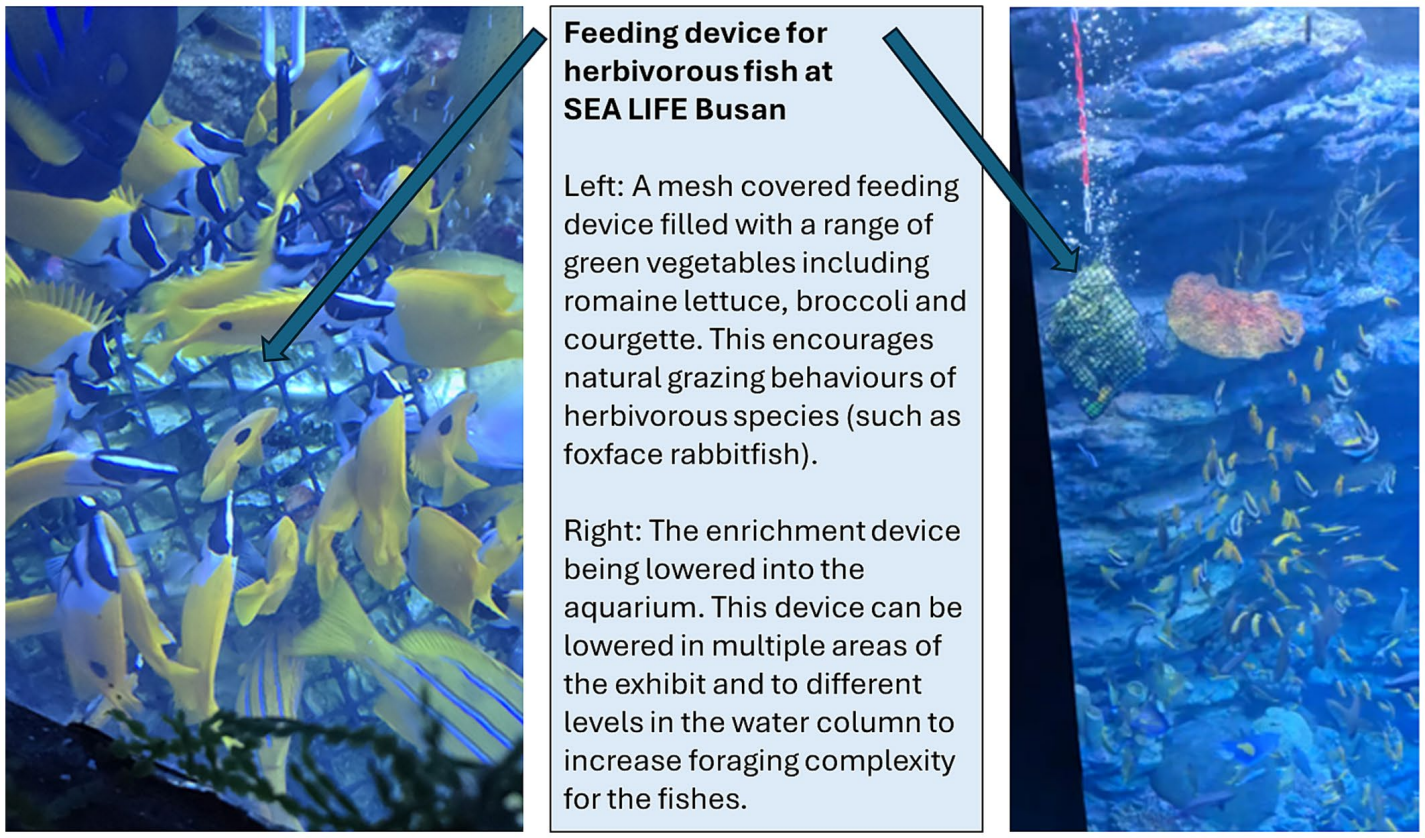
Feeder used in SEA LIFE Busan for encouraging grazing behaviour in foxface rabbitfish (*Siganus vulpinus*). Explanation of the key points of this enrichment is provided in the image. Photo credit and used with permission: Harry Jang/SEA LIFE Busan.

Food presentation also matters. Variation in food presentation can increase the need for exploration and problem-solving, while monotonous feeding can lead to reduced activity or stereotypical behaviours (96). Published zoo/aquarium nutrition research is more limited for aquatic species (when compared to terrestrial mammals, for example) but available studies do recognise that feeding format drives behavioural outcomes (97). As a practical example, staff at SEA LIFE Busan designed a feeder for providing increased grazing opportunities to herbivorous fish (Figure 1) and this increased both the amount of vegetable matter consumed and enabled wider expression of natural grazing behaviour. Such a device can be filled with naturalistic and commercial food items to increase dietary variety and be moved around an aquarium to improve exploration. This device has created a positive feeding challenge that requires environmental and social interactions more akin to wild contexts – supporting positive diversification of species-typical behaviours.

Aquatic invertebrates are also under-represented in welfare research (98), with natural diets ranging from filter feeding to active prey hunting. While specific studies on the impact of different diet types for ex situ aquatic invertebrates are scarce, promotion of species-specific feeding behaviours should be a central consideration in their care. In this way, we can ensure the conservation of important (adaptive) behaviours across ex situ generations and provide a stronger grounding for positive welfare outputs. Aquatic invertebrates sustained on simplified diets may express reduced behavioural repertoires, which likely compromises the maintenance of species-typical adaptive responses and the potential for positive welfare outputs.

Evidence-based welfare research emphasises that management inputs, including diet, should be species-specific to avoid loss of adaptive traits that contribute to positive welfare outcomes (99). Providing diets that reflect natural feeding contexts can reinforce ecologically relevant behaviours associated with species-typical foraging strategies (88, 100, 101). This may involve the provision of naturalistic food items or the presentation of commercially formulated diets in ways that encourage exploration, manipulation, and problem-solving. Consequently, dietary management extends beyond meeting nutritional requirements, influencing how animals allocate time to foraging, engage cognitively with their environment, and interact socially (102–104). Such approaches support adaptive potential and broader components of positive welfare, including resilience and opportunities for choice in environmental and social interactions. Although aquatic species are highlighted here due to their underrepresentation in the literature, designing feeding regimes that mimic natural foraging strategies should be considered across all taxa. Doing so promotes behavioural repertoires aligned with species ecology, supporting both behavioural and psychological welfare while maintaining adaptive responses relevant to species survival. Further research examining the influence of diet composition and presentation on welfare outcomes in zoo-housed animals will help align captive nutrition with ecological function, strengthening the integration of welfare, education, and conservation goals in zoological institutions.

## 3 Human impacts

### 3.1 Human-animal interactions, visitor presence and zoo animal welfare outputs

Human–animal interactions (HAIs) are a routine component of zoo environments, occurring between animals and both familiar individuals (e.g., keepers) and unfamiliar ones (e.g., visitors). With an estimated 700 million people visiting zoos annually (14), alongside daily exposure to staff, understanding how HAIs influence animal welfare is essential. Zoos are uniquely positioned to bridge the gap between humans and nature, encouraging pro-environmental behaviours essential for supporting global conservation goals (105). To meet these aims, institutions can provide a variety of HAI opportunities, ranging from observation and explanation of the animal with a member of staff, to “meet-and-greet” sessions, through to immersive experiences (potentially within the animal’s home environment) (106). HAIs also need to be evaluated within the broader institutional context of modern zoos, balancing animal welfare and conservation goals with educational outcomes and financial sustainability. While carefully designed interactions can enhance visitor learning and provide important revenue streams that support conservation and animal care (107), such potential benefits must be weighed against any welfare implications that may result in negative experiences for the animals.

The impact of HAIs varies widely depending on context, species, and individual temperament. Interactions involving zoo staff are often associated with positive outcomes, such as food provision or positive reinforcement training, which may increase activity levels (108). However, whether increased activity reflects improved welfare remains unclear and may depend on staff expertise and attitudes. Ward and Melfi (109) found that dyadic relationships can form when staff possess strong species-specific knowledge, potentially due to positive ontogenetic experiences facilitating favourable associations (110). Nonetheless, given the wide taxonomic diversity in zoos, further research is needed to determine which species are most likely to form such relationships. HAIs in reptiles, amphibians, and invertebrates remain particularly understudied, highlighting a major gap in understanding welfare impacts in these taxa (111).

Positive Reinforcement Training has the potential to strengthen human–animal relationships and positively influence animal welfare (112). However, the effectiveness of such training programmes must be empirically evaluated to remove anecdotal inference and, therefore, this requires the development, validation and application of standardised welfare measures across taxa (113). Behavioural approaches should be integrated to provide a holistic understanding of welfare (114). For example, ethogram-based behavioural assessments conducted prior to training can determine whether animals respond positively or negatively to human presence (115). When combined with species-specific qualitative behavioural assessments (QBA), these methods offer insight into emotional states through posture, movement, and behavioural expression (77), as well as motivation to engage with training sessions (116). Together, these approaches provide a more comprehensive understanding of affective states and welfare outcomes associated with HAIs, and align with previous discussion within this article on the importance of control, choice, autonomy and agency within welfare assessment frameworks (26, 81).

Contrasting with staff interactions, visitor-centred HAIs are more variable and thus more difficult to evaluate. Zoo visitor presence can be considered an indirect interaction, as the animal can experience (via species-specific sensory modalities) the presence of visitors at their enclosure and respond accordingly based on the visitors’ behaviours, or crowd size (for example). Visitor presence has been linked to positive welfare outcomes in some social species (117, 118). However, in other species, higher visitor densities (and associated change to the sound environment) increases vigilance, agitation and avoidance behaviours (119, 120). Despite this potential for negative welfare outcomes, the implications of direct visitor interactions remain broadly relatively understudied (121) and thus should be explored across more species, enclosures and facilities. Such research extensions are vital to determining how such interactions may be impacting on the ex situ roles of species, for example its contributions to conservation programmes.

Existing research suggests that handling experiences can negatively impact welfare in some species. Baird, Kuhar (122) found increased stress-related behaviours and elevated faecal glucocorticoid metabolite (FGM) levels in southern three-banded armadillos (*Tolypeutes matacus*), screaming armadillos (*Chaetophractus vellerosus*), red-tailed hawks (*Buteo jamaicensis*), and African pygmy hedgehogs (*Atelerix albiventris*) during handling. Visitor-mediated feeding experiences may offer welfare benefits in some instances, but not in others. Visitor feeding of elephants (Elephantidae), for example, has been associated with increased social interactions and reduced stereotypic behaviour compared with non-visitor feeding contexts (123). Allowing visitors to provide browse to giraffes as part of a feeding programme could encourage higher rates of browse intake, which is ultimately beneficial to their health and welfare (124), as well as providing added complexity around foraging opportunities (125). However, research has identified that zoos are not offering browse as part of such animal-giraffe interaction programmes and heightened rates of inactivity are apparent in giraffes that participate in such encounters (126).

To evaluate the welfare implications of visitor-based HAIs effectively, it is essential to first define species-specific behavioural baselines. Comparing natural behavioural repertoires observed in the wild with those expressed during in-zoo interactions (such as feeding or handling) can help identify behavioural suppression or deviation, potentially indicating compromised welfare (127, 128). Integrating time–activity budgets with additional welfare indicators, such as behavioural diversity, enables a more holistic assessment (114). Positive indicators, including play and foraging behaviours, can be used to quantify welfare outcomes (129).

However, many taxa, particularly non-mammalian species, remain underrepresented in both captive and wild behavioural research (127, 130). Establishing baseline time–activity budgets across a wider range of species is therefore essential. Such baselines would allow more accurate evaluation of how different forms of HAI (whether involving staff or visitors) affect behaviour and welfare, ultimately supporting the development of evidence-based practices that promote positive welfare outcomes in zoological settings.

### 3.2 Experiences and expectations of local zoo visitors and how the zoo provides societal benefit

With growing obligations to safeguard threatened species, zoos face increasing scrutiny and pressure to justify their societal role through demonstrable contributions to conservation, education, research, and animal welfare (2, 131, 132). Rising public awareness of conservation challenges, ethical concerns surrounding the keeping of wild animals and media exposure of poor welfare practices have intensified this scrutiny. As public values evolve, zoo visitors increasingly expect more than passive entertainment, instead seeking authenticity, transparency, and evidence that zoos actively contribute to ecological and societal wellbeing (1, 133). This shift compels zoos to reassess how they engage with visitors and communicate their purpose.

These changing expectations present both opportunities to improve and challenges to the legacy systems and infrastructures. Zoos have the potential to foster empathy for wildlife, support research that improves animal welfare, encourage pro-environmental behaviour, and contribute meaningfully to conservation. However, they often struggle to bridge the gap between institutional intent (such as conservation and welfare objectives) and visitor perceptions. Bridging this gap begins with understanding how visitors experience, interpret, and value encounters with animals and exhibits. From a visitor perspective, the implicit question is often “What’s in it for me?”, encompassing both personal benefit and broader societal value. Aligning visitor expectations with institutional goals transforms meaningful encounters into shared personal and societal impact and is essential for maintaining a zoo’s social licence to operate (SLO).

Zoos operate under legal permits but also depend on public trust and acceptance to function legitimately. In this context, SLO refers to an intangible agreement grounded in public approval, trust, and perceived ethical conduct that extends beyond minimum legal compliance (134–136). Maintaining this licence requires zoos to demonstrate that they operate ethically, responsibly, and in ways that align with societal values. As animal welfare is central to this process, modern zoos increasingly prioritise welfare at both individual and population levels, recognising its importance for conservation and education outcomes. High welfare standards are a prerequisite for public support; loss of trust may lead to reduced attendance, negative media attention, pressure to close or reform, loss of accreditation, and reduced political or financial support. Consequently, proactive management of public expectations regarding animal welfare is essential (64). Visitors expect to observe healthy, active animals in spacious, naturalistic environments that allow species-appropriate behaviour (135, 137, 138). Exhibit design strongly influences these perceptions by shaping what visitors observe, how long they engage, and how they interpret animal behaviour and welfare. Addressing visitor expectations requires visible implementation and assessment of positive welfare indicators, including opportunities for choice and control, evaluation of affective states, support for natural behaviours, engagement with relevant enrichment, and high standards of veterinary care and nutrition.

Zoos are therefore encouraged to invest further in welfare initiatives and communication while phasing out practices perceived as unethical. Compliance with local, national, and international standards, such as those established by EAZA and World Association of Zoos & Aquariums (WAZA), alongside routine welfare audits, promotes accountability and continuous improvement. Communicating these efforts is equally important. Warsaw and Sayers (135) found that although welfare accreditation positively influenced visitor perceptions to a specific zoo, many were unaware such programmes existed and this highlights the need for improved communication and visitor engagement.

Prioritising animal welfare also strengthens an institution’s credibility (139), which for zoos impacts on conservation and educational reputations. Zoos contribute through coordinated captive breeding and population management programmes, as well as through field conservation initiatives such as population monitoring and habitat restoration in partnership with local communities and NGOs. Transparent reporting enhances legitimacy by showing that animals in human care can support wild species survival goals (140). Beyond conservation, education plays a critical role in maintaining SLO. Visitor engagement strategies (including guided tours, school programmes, citizen science, immersive exhibits, and interpretive media) can foster emotional connections and promote conservation-minded attitudes (141–143). Evidence-based education provides ethical justification for keeping animals in human care and can drive social change (144). As zoos increasingly position themselves as research institutions, scientific output further supports their societal value. Research in conservation biology, veterinary medicine, animal behaviour, nutrition, and reproduction contributes to knowledge that benefits animals and ecosystems, informs best practice, and improves welfare standards (12, 20, 72, 99). For visitors, visible research activity reinforces trust by demonstrating tangible contributions to conservation and welfare. Given the propensity of behavioural research conducted within zoos (145), the presence of observers collecting data provides clear evidence to visitors of “research in action”.

Maintaining a SLO requires measurable outcomes, transparent reporting, and meaningful stakeholder engagement. By creating emotionally engaging, educational experiences that link visitor enjoyment to real-world conservation outcomes, zoos can foster public trust, inspire pro-conservation behaviour, and strengthen their societal legitimacy. The future of zoos will depend not on visitor numbers alone, but on their ability to align animal welfare, conservation impact, and visitor experience by inspiring minds in ways that meaningfully contribute to both nature conservation and the wellbeing of animals in human care.

## 4 Environmental impacts

### 4.1 The social context of translocations for population management

One of the major successes of modern zoos is the level of cooperation across institutions, countries, and continents to maintain genetically viable captive populations. Across Europe and Western Asia, EAZA coordinates EEPs for over 500 species (146). Each EEP oversees the demographics and usually the genetics of all individuals of a given species held in EAZA institutions, enabling informed decisions about breeding, non-breeding, and inter-institutional transfers. These recommendations are guided by International Union for Conservation of Nature (IUCN) principles (147) and aim to maintain healthy populations by achieving demographic targets, maximising genetic diversity, preventing skewed sex ratios, and managing issues such as accidental breeding (148). This coordinated approach enables evidence-informed translocations that are central to conservation breeding, yet such decisions rarely account for individual animal social relationships, despite their potential influence on welfare and reproductive success.

The social environment influences many aspects of animal welfare inputs and outputs. A large number of species currently subject to ex situ breeding management are social, yet the consequences of moving individuals between social groups (particularly the disruption or facilitation of social bonds) are often poorly understood (149). Importantly, in wild contexts, movement between groups is not inherently negative. For many social species immigration and emigration occur naturally as part of dispersal processes and can contribute to genetic exchange and social dynamics. However, in managed populations the timing, context and constraints of transfers may differ from natural dispersal events, potentially altering their welfare and social consequences. Socially living animals typically show clear preferences for particular group members when sleeping, foraging, resting, grooming, or otherwise interacting (150). Consistent positive interactions between individuals indicate the presence of social bonds, which underpin a group’s social structure and reflect species-specific adaptations shaped by evolutionary pressures (151). Although the adaptive value of social bonds is not always straightforward (152), there is growing evidence that the quality or quantity of social relationships can directly influence reproductive success. Stronger social bonds or greater social integration have been linked to increased reproductive rates in female feral horses (*Equus caballus*) (153), chacma baboons (*Papio ursinus*) (154), and bottlenose dolphins (*Tursiops* sp.) (155). Importantly, such effects are not limited to mammals: male wire-tailed manakins (*Pipra filicauda*) with greater social connectivity within leks also achieved higher reproductive success (156). Given that reproductive output is a key objective of captive breeding programmes, particularly for threatened species, understanding how social relationships influence welfare, integration and breeding outcomes during transfers may help align social management decisions with both welfare goals and conservation objectives. Supporting an individual’s ability to form and maintain appropriate social bonds – while recognising that dispersal or group changes may sometimes be beneficial or species-typical – may substantially improve outcomes. However, executing this in a way that promotes natural social structures and protect individual welfare requires further targeted research across species.

Although fitness benefits of social relationships can be challenging to quantify, understanding their role could also enhance welfare during translocations. Social bonds are known to buffer individuals against stress. Social buffering has been demonstrated in rhesus macaques (*Macaca mulatta*) (157), rats (*Rattus norvegicus*) (158), daffodil cichlids (*Neolamprologus pulcher*) (159) and zoo-housed Livingstone’s fruit bats (*Pteropus livingstonii*) (160). In feral horses, foals were more likely to survive following a major population reduction if a greater number of known social associates remained present (161). These findings are highly relevant to zoo management, as translocations are likely to be stressful for moved individuals, those left behind, and those in the receiving group. Moving individuals alongside close social partners may therefore improve post-transfer welfare, reproductive success, and even survival.

A key priority for zoo-based research is to identify species in which social bonds provide measurable benefits, allowing translocation guidelines to be informed not only by genetic and demographic considerations but also by fine-scale social structure. Social network analysis offers a powerful tool for this purpose, enabling identification of meaningful relationships and prediction of how removing or relocating specific individuals may affect group dynamics (149, 162). Demonstrating social buffering and other direct benefits of social bonds can further strengthen the justification for incorporating social considerations into management decisions. Finally, improving understanding of how individual social environments influence reproductive success in captivity will require coordinated, multi-institutional research, particularly studies examining both short- and long-term effects of translocations on individuals and groups.

### 4.2 Keeping the zoo wild: retaining wild-type behaviours in zoo populations and long-term breeding programmes

Zoo animals are often managed under regional population management programmes run by regional zoo membership organisation. These programmes aim to maintain ex situ populations that are demographically and genetically healthy, and can have a variety of conservation, research and education roles. Insurance populations are maintained from a sustainability perspective (163) but also as a safeguard against extinction, and this role is listed in 75% of the 103 EEPs featured on the EAZA website (146). Although most zoo animals will not be released into the wild, and conservation translocations/population restoration are only listed as a direct role in 28% of those EEPs, their insurance role assumes a possible need for such populations to be a source of animals in future conservation activities (e.g., translocations).

The sustainability of these long-term breeding programmes has been questioned, however, due to the challenges of maintaining self-sustaining, genetically diverse and behaviourally competent captive populations that can fulfil those roles (163). The IUCN guidelines for conservation translocations state that animals in source populations should show comparable morphology, physiology and behaviour to the original or remaining populations of that species in the wild (164). However, morphological differences between captive and wild animals of the same species have been recorded, such as variation in cranial shape in mammals (165), wing shape in birds (166), and head size in reptiles (167). The behaviour of captive animals can also deviate from that of their conspecifics in the wild, particularly in populations that have been in captivity for multiple generations (168). Multiple species have shown captivity-induced loss or modification of wild-type behaviours, including loss of anti-predator responses (169), altered vocalisations/social communication (170), reduced locomotion and foraging skills (171), and altered activity and feeding patterns (172). Therefore, if ex situ populations deviate from their *in situ* counterparts to the extent that they are not true representatives of the species, their role as insurance for potential future translocations will be compromised.

Zoos have enhanced collaborative efforts to address the demographic and genetic challenges of breeding programmes. For example, Global Species Management Plans (GSMP) have been developed for several species, enabling cross-region collaboration and management of *ex situ* populations that would not be sustainable with regional management alone (173). However, the behavioural competence of these populations in the long term requires additional attention. More research across taxa is needed to identify how features of the wild environment can be replicated ex situ, reducing adaptation to captivity (174) and facilitating the development of wild-type behaviours. Although ex situ research is key to assess and improve the impact of management and husbandry interventions on captive behaviour (175), increased *in situ* research efforts are also needed to better understand the natural behavioural ecology of animals and inform those interventions. Such in situ research should be planned with specific aims and methods that facilitate in situ and ex situ comparisons and to address any knowledge gaps within ex situ conservation programmes to improve efficacy (176). Furthermore, as captive and wild populations are subject to evolution and different selective pressures (177), it is important to continuously monitor wild populations to identify evolutionary changes that should be replicated in their captive counterparts so they can fulfil their conservation roles.

## 5 Institutional and reputational impact

### 5.1 The validity and usefulness of the evidence behind evidence-based concepts

An evidence-based approach involves making decisions and developing practices that are grounded in the best available scientific evidence, rather than relying solely on tradition, intuition, or anecdote (178). The integration of evidence (i.e., systematically collected data, rigorous background research, and critical evaluation of sources) develops management strategies that promote welfare, sustainability, and integrated conservation approaches (72, 99, 179, 180). However, whilst increased efforts into evidence generation is admirable, critique and appraisal of such evidence is essential to judge its quality and thus ensure that any resulting changes to animal care practice are valid and reputable improvements. Evidence can be deemed valid by assessing its scientific quality and reliability, including the use of appropriate research design (e.g., controlled experiments, replication, and randomisation), transparency of methods, robustness of sample sizes and statistical analyses, and whether results have been peer-reviewed and independently replicated (181–185). Thus, valid evidence is characterised by its credibility – in that it is relevant to the specific animal management context, being generated through methodologies that minimise bias and error.

Acknowledging that zoo-based research faces structural constraints that make achieving these criteria challenging is important. Research frequently focuses on small sample sizes due to the limited number of individuals of a given species within a single institution, and the risk of pseudoreplication can arise when individuals are housed within the same social or environmental conditions (186). Additionally, logistical limitations, ethical considerations, and institutional variability can restrict the use of controlled experimental designs. These issues are not unique to zoo science and reflect broader concerns within the life sciences regarding reproducibility and generalisability of findings (187). However, recognising these limitations is essential if the field is to advance. Multi-institutional collaboration, longitudinal monitoring, and data sharing across collections can help overcome these challenges by increasing sample sizes and improving replication, thus improving the validity and robustness of evidence generated.

Reviews of the wider literature that focus on evidence-based papers pertaining to zoo and aquarium animals show limited research interest in some taxa and areas of management (6, 9–12). Therefore, whilst evidence generation for some taxa (e.g., primates) and for some questions (e.g., behaviour) is substantial, the zoo research community needs to look more deeply at how studies on species and questions that appear more challenging to commence, or that may lack support due to limited background literature, can be encouraged, designed and executed. Reviewing how evidence is applied – both within specific elements of zoo management, such as environmental enrichment (179), and more broadly across housing and husbandry (188) – can guide future evidence gathering efforts. This process helps identify elements of current practices that are poorly evidence-based or entirely unevidenced, while also supporting the evaluation of research quality, as published methods can be critically appraised for their rigour and relevance to new species or questions.

In addition, zoos and zoo researchers should consider capturing key insights and understanding of animals that arise from the experience of animal care staff. Animal care professionals often develop a deep, experience-based understanding of the animals in their care, from repeated observations of the same individuals in a semi-systematic way (189). Such knowledge can meaningfully inform welfare assessments (190), help decipher behaviour patterns (191) and support training and handling protocols (192) enabling the development of species-specific approaches to individual elements of husbandry for the benefit of each animal. Formally capturing and integrating this practitioner-derived knowledge – what we might term “animal experience evidence” – alongside empirical data and peer-reviewed outputs enhances the comprehensiveness and applicability of management decisions. Examples of this natural science, social science and humanities integration do appear in the literature (193–195), forming a sound basis for research extension. This approach supports welfare evaluations for individual animals, and meaningful recording of their responses to in-zoo practices. It also captures the practical realities of animal behaviour, individual differences, and long-term animal–keeper interactions that are all essential considerations for the application of husbandry regimes, enclosure development and design principles and population management strategies.

Incorporating structured methods to document and evaluate such expertise (e.g., expert elicitation, structured observation records, or participatory welfare assessments) strengthens the evidence base, particularly where formal research is limited. This integration aligns with the broader principles of evidence-based practice, which advocate for combining the best available research evidence with professional expertise and contextual understanding. Keepers are “…the front-line in ensuring the highest standards of welfare for zoo animals and therefore must have a good understanding of the normal behaviour and physical ‘norms’ for each species they care for (196).” This quote beautifully encapsulates why evidence must be a two-way flow; it illustrates that keeper experiences of their zoo’s individual animals must be used to develop management strategies for the animals they know, but that quality scientific information (on the fundamentals of these species in the zoo) must be available to the keepers so they know what they are doing. Ongoing evidence generation is vital, yet its value depends on careful critique prior to implementation. Future progress may lie in developing targeted education and training that build researchers’ and practitioners’ capacity to assess the quality and applicability of evidence, ensuring that only sound and contextually appropriate findings inform in-zoo practices moving forwards.

### 5.2 Is being able to breed important? Evaluating the evidence for the benefits of breeding across zoo-held taxa

Zoos promote breeding programmes as a means to advance both conservation and animal welfare. This position assumes that reproduction is an intrinsic and fundamental biological drive, which enhances behavioural diversity, and provides opportunities for positive experiences such as courtship, mating, parental care and social bonding (197, 198). However, this assumption is rarely supported by empirical evidence. While the positive effects of good welfare on reproductive success are well established (199, 200), there is limited research examining whether reproduction itself improves individual animal welfare (198, 201). If breeding is to be justified on welfare grounds rather than population management alone, this evidence gap must be addressed.

Welfare guidelines recommend encouraging opportunities for the expression of natural behaviours, implicitly including reproductive behaviours. However, increased behavioural diversity does not inherently equate to improved welfare, and natural behaviours are not automatically beneficial (202, 203). For instance, interactions with predators are natural but not welfare positive. Reproduction similarly encompasses both positive and negative experiences, with considerable variation across species and individuals. The key knowledge gap lies in identifying the physical, behavioural, and emotional indicators associated with reproductive experiences. Addressing this would allow a shift from assumptions about “beneficial” behaviours toward evidence-based management decisions.

Reproduction may also encourage engagement in meaningful challenges that contribute to a “life worth living” (204). However, these benefits are not universal. Cronin, West (201) found no improvement in behavioural welfare indicators, such as abnormal or stress-related behaviours, in chimpanzees (*Pan troglodytes*) rearing young. Furthermore, the extent to which reproduction influences an individual’s life varies substantially between species. Schiffmann, Schiffmann (198) estimate that reproduction accounts for only 5–10% of lifetime activity in species such as Hermann’s tortoises (*Testudo hermanni*) and male jaguars (*Panthera onca*), but up to 70–95% in male cassowaries (*Casuarius casuarius*) and female red kangaroos (*Osphranter rufus*). This variation highlights the unequal potential for reproduction to affect overall lifetime welfare.

There is also a clear welfare cost associated with reproduction. In managed populations, individuals or offspring may be relocated for genetic management, potentially disrupting social bonds (205). Introductions to potential mates can involve significant risk, including aggression, injury, or death (206). During breeding seasons, competition for mating opportunities may increase stress, reduce food intake, and raise injury risk (207). Subsequent stages such as gestation, incubation, lactation, and rearing impose substantial energetic demands, representing significant physiological stress (208). While rearing young can generate positive welfare states through bonding and social learning, it also requires heightened vigilance and often reduces time available for self-maintenance behaviours such as feeding, resting, or grooming (209, 210).

One of the least quantified yet most critical welfare considerations is agency (as mentioned elsewhere in this article). Reproduction may enhance agency in some domains, particularly social interactions, but restrict it in others. In conservation breeding programmes, individuals are often paired based on genetic suitability rather than preference, limiting mate choice and competition despite their strong motivational value (211). Additionally, behaviours such as courtship, nest construction, or den selection may be constrained for management or monitoring purposes (205). While artificial nests or dens can improve safety, they may also remove opportunities for exploration, problem-solving, and skill development (212). Consequently, reproduction may simultaneously enhance and restrict agency, creating trade-offs between parental investment and other beneficial behaviours.

The lack of concrete, quantified data remains the biggest obstacle to making evidence-based decisions about the impacts of reproduction with regard to an individual animal’s welfare. We do not yet have the evidence base to determine that the positive social impacts of rearing young outweigh the negative impacts of physiological strain and mental stress, the risks of injury, or social disruption. Future best practice necessitates a nuanced, individual-by-individual approach, considering every aspect of the reproductive process, from intra-specific competition to the export of individuals, assessed for its potential positive and negative effects on welfare.

### 5.3 Advancing welfare through interdisciplinary and stakeholder collaboration

Scientists suggest that we are currently experiencing a sixth mass extinction event (213), due to the accelerating pace of climate change and its associated human impacts on the natural world (214). Thus, it has never been more crucial for zoo conservation to function effectively, with ex situ research playing a key role in supporting this objective. Traditionally, research conducted in zoo settings has faced criticism due to low statistical power and limited generalisability of findings to wider populations, including entire species (186, 215). These limitations are often driven by small sample sizes and siloed disciplinary approaches, highlighting the need for greater interdisciplinary collaboration (216).

The importance of interdisciplinary collaboration and use of knowledge is noted in several papers (217, 218). Williams, Sadler (219) describes a workshop consisting of stakeholders from companion, farm, and zoo animal sectors, focusing on the challenges and limitations of human-animal interactions, as well as the growing role of machine-animal interactions for animals under human care. A key outcome from these discussions was the recognition that further development of this research area depends on interdisciplinary collaboration, particularly to advance understanding of machine-animal interactions in ways that are both practical and centred on animal welfare.

As animal welfare science develops rapidly, emphasis is placed on integrating novel methodologies and interdisciplinary perspectives to improve welfare assessments and their outcomes (72, 220). Emerging technologies, including automated monitoring systems, integrated data platforms and artificial intelligence (AI), offer new opportunities to analyse complex behavioural and welfare datasets in zoo settings (221, 222). In addition, the adoption of technological tools and approaches has increased in zoos as a means of supporting more efficient welfare assessment, particularly in response to limitations associated with more traditional procedures (222). However, for successful implementation, collaboration across various stakeholders should be considered. Zoo scientists contribute detailed knowledge of animal behaviour, welfare indicators and biological context, ensuring that data collection and interpretation remain grounded in species-specific understanding. Data scientists and AI specialists can support advanced analytical approaches, while IT professionals and data engineers are critical for integrating and managing the data infrastructure required to support these systems (223). Such collaboration has the potential to advance the development and application of AI technologies in zoo settings (for a range of research questions and to assist zoo operations); however, achieving these advances fundamentally depends on the integration of expertise across multiple disciplines (224).

Beyond interdisciplinary research, stakeholder inclusion has also been identified as central to advancing animal welfare in zoos and aquaria. The development of effective zoo welfare strategies depends on the meaningful inclusion of a wide range of stakeholders, including animal care staff, welfare scientists, and organisational decision-makers (64). Extending this principle to zoo-based research highlights the value of engaging contributors beyond academia, as stakeholders bring diverse perspectives, expertise, and practical experience that can shape research priorities, interpretation, and application. In addition, initiatives such as the “ManyZoos” encourage collaborative research between zoological collections and partners across academic, government, and non-government sectors (216). Such approaches to research support greater transparency and trust, which are widely recognised as the key foundations of an organisation’s social licence to operate (136, 139). Collectively, interdisciplinary research and meaningful stakeholder engagement offer a pathway to advancing zoo animal welfare that aligns scientific innovation with practical application and societal expectations.

## 6 Conclusion

**Figure 2:**
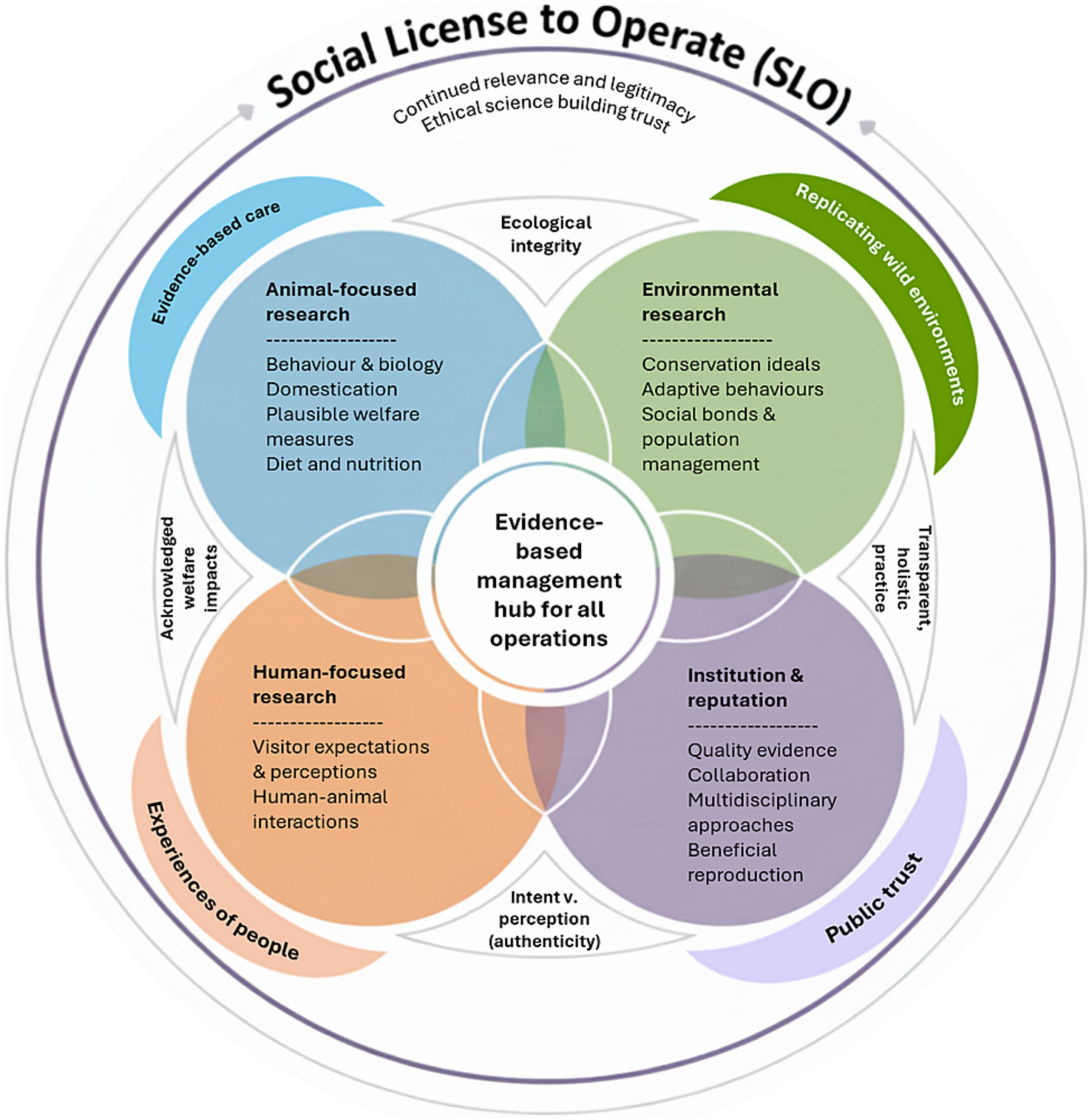
A conceptual framework illustrating how evidence-based management integrates animal-, environmental-, human-, and institution-focused research. These interconnected domains support ethical practice, ecological integrity, public trust, and transparent decision-making. Together, they underpin the social license to operate through credible, multidisciplinary evidence.

As zoos have progressed through the first quarter of the twenty-first century, this paper has examined the evolving role of zoo-based research in promoting animal welfare, and the evidence-based approach as a scaffold for their societal value and wider impacts. However, we highlight critical knowledge gaps that still constrain positive welfare outcomes, conservation credibility and engagement with wider audiences regarding the work of modern zoos. Across themes including reproduction, social structure, nutrition, human–animal interactions, behavioural integrity, and visitor engagement, a unifying challenge emerges – core management decisions are frequently informed by assumption, tradition, or logistical convenience rather than robust, species-specific and replicated evidence. A further challenge arises in determining what counts as good evidence and how this should be validated (and then applied) in the zoo. Addressing these gaps is essential if zoos are to meet rising ethical expectations and maintain their SLO. Figure 2 provides a visual representation of the interlinked components of the key themes of our discussion.

Key questions recur throughout this work. Does reproduction enhance individual welfare, and under what conditions do its benefits outweigh any physiological and social costs? Alongside, what are the long-term costs of keeping animals in captivity to achieve such conservation or population sustainability reproduction goals? How do social bonds influence welfare, stress resilience, and reproductive success, and how should these relationships inform translocation decisions? To what extent do captive diets support not only physical health but behavioural and cognitive needs? How do interactions with keepers and visitors affect animals across taxa, and which forms of engagement support rather than compromise welfare? Finally, how can zoos demonstrate measurable conservation, educational, and welfare outcomes in ways that are transparent and meaningful to the public?

Answering these questions requires a shift toward coordinated, multi-institutional and open research frameworks that enable adequate sample sizes, longitudinal monitoring, and cross-species comparison. Standardised welfare assessment tools, grounded in validated behavioural and physiological indicators and informed by wild baselines, are essential. Integrating social network analysis, controlled intervention studies, and emerging technologies for behavioural monitoring will allow zoos to move beyond descriptive studies toward predictive (potentially automated), management-relevant science. Use of modelling approaches and harnessing the power of AI and machine learning to execute big data projects relevant to the aims of the zoo could bolster the impact of research. Equally important is embedding research outcomes into operational decision-making, ensuring findings directly inform breeding recommendations, enclosure design, nutrition, and visitor programmes.

Operationally, such an approach offers clear benefits: improved welfare and health outcomes, more successful breeding and translocations, reduced management risk, and more efficient use of resources. Strategically, it strengthens public trust by demonstrating that zoos are evidence-driven institutions capable of delivering measurable benefits for animals, conservation, and society. Ultimately, the future legitimacy of zoos will partly depend on their ability to integrate rigorous science with ethical practice, ensuring that every aspect of animal management is justified, transparent, and aligned with their stated purpose (e.g., conservation, education, engagement).

Ensuring practices are justified ultimately relies on the evidence-based approach. The contemporary scientific literature offers an unprecedented volume of published research, yet not all evidence carries equal credibility or applicability. An expansion of open-access publishing has increased accessibility but has also facilitated the emergence of predatory journals. Consequently, publication alone should not be taken as confirmation of evidential reliability. Even within reputable journals, individual studies must be critically evaluated. Findings based on small sample sizes, short study durations, or highly specific contexts have value but must be evaluated and appraised in context. Individual animal variation and institutional conditions differ, and thus a case study’s results will be within the perspective of the study zoo itself. Such research outputs can become credible evidence when they are characterised by methodological transparency, appropriate statistical power, replication across settings, and convergence with existing research. Publication bias can further complicate interpretation, as positive results are more likely to be published than neutral or negative findings, potentially overstating the effectiveness of interventions. Practitioners must therefore assess whether conclusions are proportionate to data presented and whether limitations are explicitly acknowledged. Developing a culture of critical appraisal, where evidence is questioned, validated, and tested before adoption, is essential to ensure that scientific research reliably informs ethical, effective, and welfare-positive practice moving forward.
